# The Emergence of Behavioral Testing of Fishes to Measure Toxicological Effects

**DOI:** 10.5487/TR.2009.25.1.009

**Published:** 2009-03-01

**Authors:** Janie S. Brooks

**Affiliations:** 12 Innovative Drug Research Center for Metabolic and Inflammatory Disease, College of Pharmacy and Research Institute of Pharmaceutical Sciences, Seoul National University, 599Gwanangno, Gwanak-gu, Seoul, 151-742 Korea; 22 Division of Science and Mathematics, Brevard College, Brevard, Brevard, North Carolina 28712 USA

**Keywords:** Zebrafish, *Danio rerio*, Behavior, Toxicology, Operational definitions

## Abstract

Historically, research in toxicology has utilized non-human mammalian species, particularly rats and mice, to study *in vivo* the effects of toxic exposure on physiology and behavior. However, ethical considerations and the overwhelming increase in the number of chemicals to be screened has led to a shift away from *in vivo* work. The decline in *in vivo* experimentation has been accompanied by an increase in alternative methods for detecting and predicting detrimental effects: *in vitro* experimentation and *in silico* modeling. Yet, these new methodologies can not replace the need for *in vivo* work on animal physiology and behavior. The development of new, non-mammalian model systems shows great promise in restoring our ability to use behavioral endpoints in toxicological testing. Of these systems, the zebrafish, *Danio rerio*, is the model organism for which we are accumulating enough knowledge *in vivo*, *in vitro*, and *in silico* to enable us to develop a comprehensive, highthroughput toxicology screening system.

### *Prevalent methods of toxicological testing*

Toxi- cology, the branch of science dealing with poisons and their effects, has a long history dating back to the work of Paracelsus in the sixteenth century and Orfila in the late eighteenth/early nineteenth centuries (for a review of the early history of toxicology see Langman and Kapur, 2006). Overall, toxicologists have a three-fold mission (1) to characterize toxins chemically and physically, (2) to elucidate how toxins create harmful effects in biological targets, and (3) to develop better detection and quantification techniques for identifying the presence of toxins in the environment. Scientists in the field take a multi-system approach in the examination of effects, from the cellular level, to individual organisms, to biological communities, and beyond. Studies in toxicology can be grouped into three general categories based on the experimental setting for the biological material being tested: *in vivo*, *in vitro*, or *in silico*.

***In vivo techniques:*** Perhaps the oldest of experimental settings, *in vivo* experiments measure effects / processes in the living body of an organism or in a real-life situation (e.g., behavioral and ecological studies). Although early work in the field focused on humans through the examination of human cadavers, *in vivo* research now predominantly involves the use of non-human model species: similarities in morphology and physiology between several mammalian species (e.g., guinea pigs, rabbits, rats, and mice) and humans allows extrapolation of model species effects to estimate human effects, including lethality, embryonic development and teratology, and behavior and learning problems. While *in vivo* studies remain valuable in toxicological screening and research, ethical considerations and exponential increases in the number of chemicals needed to be screened have led to a shift away from *in vivo* testing (Meyer, 2003).

***In vitro techniques:*** An increasing need for non-invasive, higher-throughput screening has led to the increasing investment of research effort and money into the development of *in vitro* tests and model systems. Although usage varies slightly among toxicologists, the term *in vitro* refers to experimentation conducted on materials outside the living body (e.g., cell culture) or in artificial situations. Within this area of toxicological

**Table 1.** Examples of *in vitro* tests in use or in development for toxicological testing*

| Use | Acronym | Description |
|---|---|---|
| Eye irritancy | HET-CAM<br>BCOP<br>IRE<br>ECE | Embryonated chicken egg<br>Isolated bovine cornea<br>Isolated rabbit eye<br>Enucleated chicken eye |
| Phototoxicity | 3T3 NRU PT | Neutral red uptake in 3T3 mouse fibroblast cells |
| Skin corrosivity | Epiderm, EPISKIN<br>TER | Reconstructed human skin<br>Rat skin transcutaneous electral resistance |
| Embryotoxicity teratogenicity | WEC<br>MM<br>ESC | Whole embryo culture of rat<br>Chick cardiomyocyte micromass system<br>Embryonic stem cell tests |
| Sensitization | LLNA | Local lymph node assay<br>Induction of IL-1β production in human dendritic cells |
| Nephrotoxicity | | Transepithelial resistance and insulin permeability |
| Neurotoxicity | | Genetically engineered neuronal cell lines |
| Epithelial barrier damage | | Fluorescein leakage test |
| Carcinogenicity | SHE Balb/c- 3T3<br>BigBlue, Mutamouse | Co-culture of Syrian hamster embryo and Balb/c-3T3 cells<br>Transgenic cell lines for either LacI or LacZ, respectively |
| Genotoxicity | Micronucleus<br>Comet<br>3D<br>Ames II Mix<br>Vitotox | DNA fluorescent probe of cell micronuclei<br>SCGE (single cell gel electrophoresis)<br>Damaged DNA detection using plasmid DNA as a substrate for *in vitro* repair reaction<br>DNA mutagenesis assay<br>GMO bioluminescent *Salmonella typhimurium* |
| Higher-level Interactions | | 3D organotypic and reconstructed tissue cultures |

*Based on information in the following reviews: Curren *et al*., 1997; Marzin, 1999; Carere *et al*., 2002; Liebsch and Spielmann, 2002; Bhogal *et al*., 2005; Hoffmann and Hartung, 2006.

research, there are two important aims: to develop screening tests that can replace accepted *in vivo* tests (Table 1) and to elucidate molecular and physiological mechanisms to explain how effects are generated from toxic exposure.

The first focus of *in vitro* research is toxicological screening. Validated tests would allow us to more fully move away from the use of animals for routine, bulk screening of chemical candidates. These tests employ cell cultures and tissues of various origins (Table 1). A concurrent effort is being made to identify biomarkers for physiological processes impacted negatively by chemical exposure; for example, glutathione S-transferase (GST) levels can indicate hepatotoxicity and alanine transaminase levels can indicate liver injury (Shaw, 2005). However, any putative *in vitro* test must go through a rigorous approval processes with various governmental regulatory agencies before it can substitute for an existing screen (Liebsch and Spielmann, 2002; Meyer, 2003; Hoffmann and Hartung, 2006). Four tests have received European Union validation to replace the Draize eye test for eye irritancy: ones utilizing embryonated chicken egg (HET-CAM), isolated bovine cornea (BCOP), isolated rabbit eyes (IRE), and enucleated chicken eyes (ECE) (Liebsch and Spielmann, 2002). In addition, Epiderm™ and EPISKIN®, both forms of reconstructed human skin, have been approved as screens for skin corrosivity. Most of the other proposed *in vitro* screening methods are still in development or in the validation process (e.g., sensitization measured by the induction of interleukin-1b production in human dendritic cells; Carere *et al*., 2002).

Going beyond toxicological screening, the second aim of researchers is to understand the mechanisms by which chemical exposure develops into toxicological effects. The development of cell cultures of primary, immortalized, and genetically-engineered cell lines allows us to study the effect of exposure on basic molecular processes: signal transduction pathways, biochemical pathways, gene regulation, cell signaling, etc. An attempt to describe the advances made in these lines of research is beyond the scope of this review. Even with the rapid rate of discovery made possible through *in vitro* methods employing cell culture, there are limitations to this approach. Primarily, cell cultures are missing the biological sophistication of *in vivo* systems. Cell culture, especially monoculture, lacks the higher-level system interactions found in tissues and organs (Hoffmann and Hartung, 2006). New *in vitro* approaches show promise in overcoming these limitations: co-cultures reconstruct intercell interaction and communication, micromass culture reproduces the three-dimensional structure found in tissue, and tissue/organ culture preserves both three-dimensional structure and the interplay of cell types (Carere *et al.*, 2002).

Along with our greater understanding in molecular and cellular toxicology comes advances in toxicogenomics, the application of genomic techniques to toxicology. Toxicogenomics can be divided into three areas: transcriptomics, comparison of gene expression as measured by DNA or RNA microarrays; proteomics, comparison of the proteome as measured through gel or solid phase techniques; and metabonomics, comparison of metabolites in tissues, plasma, or urine (Battershill, 2005; Chen, 2007). The vast amounts of information generated by these genomic techniques lead to increased application of bioinformatics, the use of computers to process, analyze, and store these data sets.

***In silico* techniques:** From our knowledge gained from *in vivo* systems, *in vitro* systems, and toxicogenomics, *in silico* research has emerged as a new experimental approach to toxicological testing. *In silico* studies try to predict toxicological effects of new chemicals and chemical mixtures based on mathematical models. One model paradigm is the quantitative structure-activity relationship method, QSAR: this method predicts the toxic effect of a new compound based upon the chemical structure and functional groups of the chemical (Simon-Hettich *et al.*, 2006). QSAR models currently in development are DEREK (Deduction of Risk from Existing Knowledge) and CASE (Computer Automated Structure Evaluation) (Bhogal *et al.*, 2005). These models can be used when toxic effects can be attributed specifically to certain physical and chemical properties of a structure. An alternative paradigm is the physiologically based biokinetic model, PBBK (Simon-Hettich *et al.*, 2006; El-Masri, 2007). This method combines results from literature (bioinformatics, physiology, biomarkers) with predictive computation techniques to estimate possible effects of exposure not only to single compounds but also to mixtures. Current *in silico* methods are still in development and have yet to complete validation procedures.

### *Re-emergence of behavioral testing*

Regardless of the advances we make in the development of alternative methods, there remains a need for *in vivo* and whole animal *in vitro* testing. It is not enough to increase knowledge of molecular and cellular processes, we must also increase our understanding of the impacts of toxic exposure on the higher level biological processes operating in individuals, populations, and biological communities. How do toxicants effect interactions of organ systems within an individual, of individuals with their environments, of individuals within a population, and of different species' populations within the biological community? Any attempts to develop more comprehensive, integrated testing strategies must take these interactions into account (MacPhail, 1992; Tilson, 1993; Hoffmann and Hartung, 2006). Behavioral testing is re-emerging as a focus of toxicological method development, as behavioral experiments provide a means of approaching these questions.

"Behavior represents the unique interface between

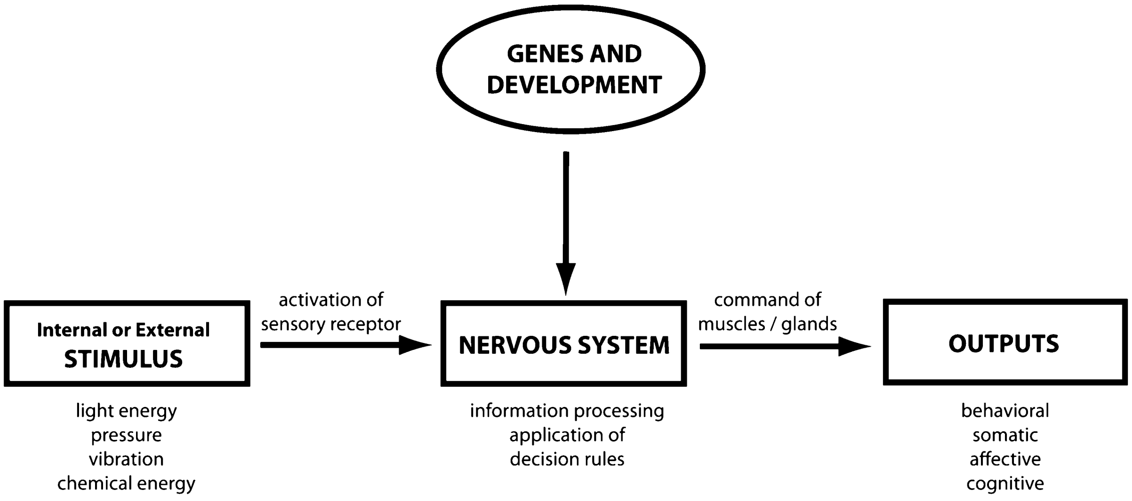

**Fig. 1.** Behavior is the integrated response of an organism to stimuli in its environment.

intrinsic and extrinsic forces that determine an organism's health and survival." (MacPhail *et al.*, 2009) Behavior is an output of the nervous system that allows an organism to respond to stimuli in the internal and external environment (Fig. 1). Limits on behavioral phenotype are imposed both by the individual's genotype and by neural development, which in turn is affected by genotype and by environmental events (White *et al.*, 2007). Behavior greatly impacts the fate of an organism in its environment, especially those behaviors dealing with life history tasks (*i.e.*, traits that influence survival and reproduction, such as feeding behaviors, mating behaviors, and parental care behaviors). Behavior results from the integration of cellular, molecular, physiological, and neurological processes (MacPhail, 1992); therefore, toxicological exposure that alters these processes can result in observable changes in behavior (Kulig *et al.*, 1996).

In fact, behavior may be more sensitive than other indicators of toxicological impact (Tilson, 1993). In other words, behavioral effects can sometimes be observed at lower levels of exposure than those required to induce teratogenic, mutagenic, or carcinogenic effects. Conversely, some toxicological effects measured through *in vitro* methods or predicted by *in silico* models may not actually alter behavior or other life history characteristics; therefore, those effects would not impact the outcome of an individual's interaction with the environment (i.e., would not result in changes to Darwinian fitness or inclusive fitness of the effected individuals). Behavioral testing allows us to better understand the ecological relevance of toxicological effects.

***Behavioral testing methodology:*** A behavioral testing system used to identify toxicological effects cannot reply upon casual observation, but rather it must employ systematic methodology that allows reliable, reproducible, quantifiable data collection. Behavioral studies must be able to measure not only major impairment resulting from exposure, but also subtle dysfunction. Behavioral toxicologists utilize methods already developed by researchers in the fields of behavioral ecology and biopsychology (Kulig *et al.*, 1996; Alcock, 2005; White *et al.*, 2007). Some of the features of a robust testing system include the development of operational definitions, the standardization of testing conditions, and the sampling of large numbers of individuals.

Reproducible measurement of behaviors is achievable through the use of operational definitions, which define behaviors in objective, concrete terms without invoking intention, purpose, or mental state (Renner and Renner, 2005). Clear operational definitions allow quantification of behavior, create interobserver consistency, and facilitate comparisons among different treatment groups (Glover, 2003; Renner and Renner, 2005; Martin and Bateson, 2007; White *et al.*, 2007). In the broad spectrum of possible animal activities, ethologists separate behaviors into two general categories: states and events. States are considered to be the broader categories of activity (*e.g.*, foraging, flying, resting); events consist of the points of change from one state to another (*e.g.*, walking to sitting) or a specific behavior that occurs within the more general state (*e.g.*, prey capture). The goal of creating operational definitions is to provide the observer with concrete guidelines for assigning behavioral data to specific, unambiguous categories: the criteria defining the beginning and the end of states and the criteria for event occurrences. With modern recording techniques, researchers can perform continuous sampling of large groups of individuals; computer programs like JWatcher (UCLA and Macquarie University) and Ethovision® (Noldus Information Technology) can be used to process the recordings. In using these analysis programs, researchers employ operational definitions, even if unknowingly: the program parameters and filters set by the operator define what data will be processed as behavior by the program (*e.g.*, an input filter defining the minimum distance required to begin tracking locomotion).

In addition to operational definitions, the use of behavioral testing in toxicological research is aided by standardization of husbandry and experimental procedures. Standardization helps to reduce variation from various sources, including the environmental influence on behavioral expression and the existence of biological circadian rhythms. As stated earlier, an organism's environment exerts great influence on behavioral expression: environmental conditions and events not only affect gene expression during embryonic and juvenile development, but also the environment provides the background context in which stimuli are perceived. Variation in behavior is also due to the normal circadian rhythms seen in biological processes. Circadian rhythms can be observed in hourly/daily patterns of the magnitude of performance of a behavior and of the degree of population variation (MacPhail *et al.*, 2009). Careful experimental design can minimize the influence of these sources of variation on the behaviors measured, increasing the power of the study to identify the effects of toxic exposure. Numerous references provide guidance on experimental design for ethological studies (Kulig *et al.*, 1996; Ploger and Yasukawa, 2004; Martin and Bateson, 2007).

Finally, testing large numbers of individuals is crucial for behavioral studies. Even in the most carefully controlled studies, one will observe individual variation in behavior. The inclusion of large numbers of subjects allows behaviorists to better understand the population level of behavior: the range of expression in behavioral phenotype existing in population, the average behavioral expression (the "mean" phenotype) found in the group, and the magnitude of variance present among individuals. With knowledge of these population characteristics, a researcher increases the number of possible endpoints to measure for evidence of toxic effects. Not only can the effect of exposure on individual behavior be measured, but also the effects of toxins on the behavior of populations can be measured.

### *Danio rerio as a model organism for toxicological research*

Previously, the standard model species used for behavioral toxicology were non-human mammals, particularly mice and rats; the close phylogenetic relatedness and similarities in morphology and physiology between rodents and humans facilitate the use of rodent data to draw conclusions about human impacts. However, employing mammalian models places limits on the scope and size of toxicological studies (Hill *et al.*, 2005; Lieschke and Currie, 2007). Disadvantages of mammalian model systems include a high requirement for staff and infrastructure to maintain animal husbandry, high annual per animal expense, increasing oversight and legal restrictions on experimental protocols, and complications caused by mammalian reproductive biology such as internal fertilization, intrauterine development, long gestation time (compared to invertebrates and fishes), small litter sizes and postnatal care. Moreover, with mammalian species it is difficult to obtain the large sample sizes needed for ethological testing and high throughput screening of the ever increasing number of candidate compounds.

In response to the need for a model system geared for high throughput screening, researchers are exploring the potential of the zebrafish (*Danio rerio*) as a new model vertebrate system for pharmaceutical and toxicological studies (Dowling, 2002; Guo, 2004; Hill *et al.*, 2005; Lieschke and Currie, 2007; Peterson *et al.*, 2008). Even though there is greater phylogenetic distance between fish species and humans, conservation of genomic, biochemical, and physiological processes across the vertebrate taxa (indeed, throughout Kingdom Animalia) will still allow us to use zebrafish data to infer the impact of exposure on humans. The features of the reproductive biology of zebrafish are ideal for experimental study and manipulation: external fertilization, external development, rapid development, and high fecundity. For embryos, their small size, chemical permeability and optical transparency facilitate observation and experimental manipulation. There is a lower cost of husbandry involved in maintaining fish cultures, and the best breeding and maintenance conditions have already been determined (Westerfield, 2000). With the high fecundity of this species, larger sample sizes and high throughput techniques are possible. Much is already known about this species' biology, including morphology, biochemistry, physiology, development, and sex / age effects on those processes; therefore, toxicologists have a good baseline for comparison when looking for evidence of toxic exposure.

***Genomic work on zebrafish:*** Initially, zebrafish were used as a model species for work in developmental biology and molecular genetics. From this work, an extensive zebrafish genomics "toolbox" has been developed, with techniques in forward genetics (chemical and

**Table 2.** Online resources for zebrafish (*Danio rerio*) research

| Description | Website (February 2009) |
|---|---|
| ZFIN: The Zebrafish Model Organism Database | http://zfin.org |
| ZIRC: Zebrafish International Resource Center | http://zebrafish.org/zirc/home/guide.php |
| The Zebrafish Book (Westerfield, 2000) | http://zfin.org/zf_info/zfbook/zfbk.html |
| Zebrafish a peer-reviewed journal | http://www.liebertonline.com/loi/zeb |
| The *Danio rerio* Sequencing Project | http://www.sanger.ac.uk/Projects/D_rerio |
| Trans-NIH Zebrafish Initiative: | http://www.nih.gov/science/models/zebrafish/ |
| ZF-MODELS: Zebrafish Models for Human Development and Disease | http://www.zf-models.org |
| Ensembl zebrafish genome assembly version 7 (Zv7) | http://www.ensembl.org/Danio_rerio |
| NCBI Zebrafish Genome Resources | http://www.ncbi.nlm.nih.gov/genome/guide/zebrafish |
| WashU-Zebrafish Project | http://zfish.wustl.edu/ |
| Tübingen zebrafish stockcenter | http://www.eb.tuebingen.mpg.de/core-facilities/zebrafish-stockcenter/tubingen-zebrafish-stockcenter/ |
| TIGR Zebrafish Gene Index | http://compbio.dfci.harvard.edu/tgi/cgi-bin/tgi/gimain.pl?gudb=zfish |
| NIH Zebrafish Gene Collection | http://zgc.nci.nih.gov/ |

insertional mutagenesis), reverse genetics (morpholines and targeting-induced local lesions in genomes (TILLING)), expression profiling (gene chip, spotted microarrays), genomic sequencing, physical genetic maps, mutant collections, cDNA collections, and transgenics (Zon and Peterson, 2005; Aleström *et al.*, 2006). In addition, primary and immortal cell lines for both embryonic and adult tissues have been established. The vast amount of known information on zebrafish genetics is easily accessible on the Internet (Table 2).

***Behavioral studies on zebrafish:*** Zebrafish behavior shows great potential for use in toxicological screening assays (Orger *et al.*, 2004; MacPhail *et al.*, 2009; Miklósi and Andrew, 2006). In order to fully use behavioral endpoints to measure toxicity, we must have a better understanding of the basic behavioral repertoire of this species. Work on characterizing the behavior of this species is accelerating. Ethologists have created descriptions of 'natural' behavioral patterns like innate and learned preferences, phobias, species-specific patterns for feeding and escape, territoriality and mate choice, behavioral lateralization, and boldness (Miklósi and Andrew, 2006). Researchers have also studied the development of sensory and motor function (Easter and Nicola, 1996; Saint-Amant and Drapeau, 1998); reflexes, learning, and memory (Kimmel *et al.*, 1974; Levin *et al.*, 2004; Orger *et al.*, 2004; Zeddies and Fay, 2005;); locomotion (Budick and O'Malley, 2000; Drapeau *et al.*, 2002; Burgess and Granato, 2007; Brooks *et al.*, 2008; MacPhail *et al.*, 2009); the escape response (Weber, 2006); habituation (Best *et al.*, 2008); and the behavioral effects of ethanol exposure (Lockwood *et al.*, 2004). While the volume of information on the behavior of the zebrafish does not yet approach that of the behavior of mammalian model systems, our understanding of the behavioral repertoire of the zebrafish will grow exponentially as more ethologists, pharmacologists, and toxicologists study this fascinating species, allowing us to identify more behavioral endpoints for toxicological screening.

### *Concluding remarks*

With the use of *Danio rerio* as a model system for toxicological research, a truly comprehensive, integrated testing program is achievable: the incorporation into a single methodology the *in vivo* knowledge of basic zebrafish biology, development, physiology, and behavior; *in vitro* knowledge of molecular and cellular processes through the use of molecular biology and cell culture techniques; and *in silico* capability made possible through use of the zebrafish genomics knowledgebase. Work remains to be done on method development and validation, but the zebrafish model system holds great promise for the creation of a comprehensive, high throughput, toxological screening system.

## ACKNOWLEDGMENTS

Dr. Brooks received support from a postdoctoral fellowship in the Brain Korea 21 Program of the Korean Ministry of Education and from a Korea Science and Engineering Foundation (KOSEF) grant funded by the Korean government (MEST) (No.R11-2007-107-01001-0).
